# Plasma Metabolome Alterations Discriminate between COVID-19 and Non-COVID-19 Pneumonia

**DOI:** 10.3390/metabo12111058

**Published:** 2022-11-02

**Authors:** Tushar H. More, Bahareh Mozafari, Andre Märtens, Christian Herr, Philipp M. Lepper, Guy Danziger, Thomas Volk, Sabrina Hoersch, Marcin Krawczyk, Katharina Guenther, Karsten Hiller, Robert Bals

**Affiliations:** 1 Department of Bioinformatics and Biochemistry, Braunschweig Integrated Centre of Systems Biology (BRICS), Technische Universität Braunschweig, 38106 Braunschweig, Germany; 2 Department of Internal Medicine V-Pulmonology, Allergology and Critical Care Medicine, Saarland University, 66421 Homburg, Germany; 3 Department of Anesthesiology, Intensive Care Medicine and Pain Therapy, Saarland University, 66421 Homburg, Germany; 4 Department of Internal Medicine II-Gastroenterology, Saarland University, 66421 Homburg, Germany; 5 Helmholtz Institute for Pharmaceutical Research Saarland (HIPS), Helmholtz Centre for Infection Research (HZI), Saarland University Campus, 66123 Saarbrücken, Germany

**Keywords:** COVID-19, non-COVID-19 pneumonia, metabolomics, metabolic profiling, multivariate statistics, machine learning, plasma, mass spectrometry, community-acquired pneumonia, system biology

## Abstract

Pneumonia is a common cause of morbidity and mortality and is most often caused by bacterial pathogens. COVID-19 is characterized by lung infection with potential progressive organ failure. The systemic consequences of both disease on the systemic blood metabolome are not fully understood. The aim of this study was to compare the blood metabolome of both diseases and we hypothesize that plasma metabolomics may help to identify the systemic effects of these diseases. Therefore, we profiled the plasma metabolome of 43 cases of COVID-19 pneumonia, 23 cases of non-COVID-19 pneumonia, and 26 controls using a non-targeted approach. Metabolic alterations differentiating the three groups were detected, with specific metabolic changes distinguishing the two types of pneumonia groups. A comparison of venous and arterial blood plasma samples from the same subjects revealed the distinct metabolic effects of pulmonary pneumonia. In addition, a machine learning signature of four metabolites was predictive of the disease outcome of COVID-19 subjects with an area under the curve (AUC) of 86 ± 10%. Overall, the results of this study uncover systemic metabolic changes that could be linked to the etiology of COVID-19 pneumonia and non-COVID-19 pneumonia.

## 1. Introduction

The coronavirus disease 2019 (COVID-19) pandemic has been the primary health concern affecting millions of human lives worldwide in the last two years. The illness, which began with a series of pneumonia cases of unknown etiology, was soon confirmed to cause severe acute respiratory syndrome [1,2,3]. COVID-19 primarily affects the respiratory system and can progress into a life-threatening systemic illness with organ failure [4]. The majority of COVID-19 cases are either asymptomatic or with minor symptoms, while 14% develop severe symptoms, including pneumonia and acute respiratory distress syndrome (ARDS), of which 5% of critical cases lead to 2.3% fatality [5]. The primary clinical manifestation of a COVID-19 infection is pneumonia, a common acute respiratory infection involving the alveolar and distal bronchi of the lungs [6]. Community-acquired pneumonia (non-COVID-19 pneumonia) is an important disease entity, which is frequently caused by bacterial pathogens and results in significant morbidity and mortality [7,8]. Ever since the onset of COVID-19, attempts have been made to compare the clinical features of COVID-19 pneumonia with non-COVID-19 pneumonia, which could help in distinguishing COVID-19 pneumonia from other respiratory diseases [9,10,11,12].

It is now known that COVID-19 has different radiological features than non-COVID-19 pneumonia [13,14,15]. However, limited information is available concerning other clinical characteristics of COVID-19 disease [9,10,12]. As inflammation caused by pneumonia affects pulmonary blood circulation, biochemical changes might also be detectable in the blood. Details on the disease mechanisms of COVID-19 have been uncovered using genomic, transcriptomic, and proteomic approaches [16,17,18,19,20,21,22,23,24,25,26,27]. However, limited studies have investigated metabolomic changes with a particular emphasis on COVID-19 disease and non-COVID-19 pneumonia [28,29,30]. Metabolomics offers a unique advantage for the characterization of biochemical events influenced by COVID-19 disease. Some studies have indeed characterized the COVID-19-metabolome within the context of clinical features, cytokine levels, and disease severity [31,32]. However, the impact of pulmonary pathophysiology on the plasma metabolome has not yet been investigated in detail. Additionally, the blood concentrations of inflammatory mediators are altered in COVID-19 and linked to pathomechanisms and clinical outcomes [33,34].

The aim of this study was to characterize the blood metabolome of patients with severe COVID-19 pneumonia and non-COVID-19 pneumonia. We report the metabolic investigation of plasma samples in subjects with COVID-19 disease, pneumonia, and controls. We also compare venous and arterial plasma samples from the lung artery and the lung vein to elucidate the metabolic differences that may result from lung inflammation. Furthermore, we compare transpulmonary gradients of different inflammatory cytokines between the three patient groups. Finally, we correlate metabolic changes with measured cytokine levels in these subjects and identify potential markers for disease severity.

## 2. Materials and Methods

### 2.1. Subject Recruitment and Sampling

The present work used the baseline data and follow-up data of the COVID-19 cohort CORSAAR (*n* = 43) and its control cohort PULMOHOM, which are multi-center studies focusing on pathomechanisms and the role of risk factors in COVID-19 and other inflammatory lung diseases. Within the PULMOHOM cohort study, 23 patients with non-COVID-19 pneumonia and 26 control patients undergoing elective non-pulmonary surgery have been included. The inclusion criteria for this non-pulmonary, surgical cohort were planned surgery, no known pulmonary disease, male sex, central venous line (CVC), and peripheral arterial line (PAL) placement during surgery. We chose patients undergoing surgery because a central venous catheter and an arterial catheter were placed as routine measures and allow for obtaining blood without further invasive procedure. Patients for the COVID-19 cohort were included within 3 days of admittance to the hospital and recruited at the Saarland University Hospital Homburg, the Caritas Hospital Saarbrücken (St. Theresia and St. Josef), the Hospital Saarbrücken (Winterberg), and the SHG-Hospital Völklingen. The studies have been approved by the ethics committee of the Medical Council of the Saarland (Ethikkommission der Ärztekammer des Saarlandes, 62/20), and all patients or their legal representatives gave their informed consent. Basic and anthropomorphic characteristics and vital parameters were assessed based on measurements, questionnaires, and standardized interviews; basic data are shown in Table 1. There were no differences in age and BMI between the groups (ANOVA testing with Bonferroni post-hoc testing), only the COVID-19 group included female patients. The COVID-19 group comprised 33 hospitalized patients on normal care units, 8 ventilated patients on the ICU, and one outpatient. From the hospitalized patients, 10 patients died in the course of the disease. All COVID-19 patients were recruited during the first wave of COVID and therefore were infected most likely by the alpha variant (B.1.1.7). Information regarding comorbidities of the study cohort is included in Supplementary Table S1. Detailed characteristics of all COVID-19 patients is included in Supplementary Table S2.

Approximately 20 mL of blood was drawn from CVC and PAL: 2 EDTA (ethylenediaminetetraacetic acid) tubes with 5 mL, 2 serum tubes with 10 mL, and 2 RNA-PAX gene^®^ tubes with 5 mL. We then centrifuged the samples from the EDTA and serum tubes in the Beckmann Coulter type Allegra X-30R Centrifuge. The EDTA samples were centrifuged at 2500 *g* for 20 min at 20 °C. The serum tubes had to be allowed to clot for 30 min before centrifugation and the samples could then be centrifuged at 1300 *g* for 10 min at 20 °C. EDTA and serum were pipetted off immediately after centrifugation. In the case of the EDTA samples, the supernatant and the serum were frozen at −80 °C and stored. Only the supernatant of the serum samples was frozen. The subsequent sample transport to the biomaterial bank took place on dry ice.

### 2.2. Measurements of the Blood Concentration of the Cytokines

The samples were evaluated using the multiplex cytokine array from Myriad (City, USA). Until the test was carried out, all samples were stored under −70 °C. A part (aliquot) of each sample was added to individual multiplexes of the selected MAP (multi-analyte profile) and to a blocker. Different assays were used for the different cytokines. The Human Inflammation MAP^®^ v 1.1 was used for the inflammatory biomarker and was therefore used for the evaluation of most of the cytokines in our study.

In addition, special Custom Maps^®^ were created in order to test individual cytokines that would otherwise be distributed over several multiplexes. These included the cytokines AXL, HCC-4, FAS, HGF, TRAIL-R3, AFP, CA-125, CA-19-9, CEA, hCG, NSE, MMP-1, MMP-7, MMP-9 total, ANG -1, CA-9, Decorin, IL-18bp, PECAM-1, and SP-D.

### 2.3. Metabolite Extraction

The plasma samples were thawed on the ice for 30 min prior to extraction. Plasma sample containing 11 µL was mixed with 100 µL of an ice-cold extraction solvent (methanol/water, 8/1, −20 °C) that contained 2 µg/mL of D6-glutaric acid and U^13^C-ribitol as internal standards. Subsequently, the mixture was vortexed at (1400 rpm, 4 °C, 10 min) and centrifuged at (13,000 *g*, 4 °C, 10 min) to precipitate proteins and extract metabolites. The supernatants (90 µL) were transferred to glass vials, evaporated to dryness at 4 °C using speed-vac, and stored at −20 °C until gas chromatography-mass spectrometry (GC-MS) measurement. Pooled quality control samples were prepared by mixing 10 µL of plasma from each sample and sample pools were extracted using the aforementioned steps.

### 2.4. GC-MS Measurements

Before GC-MS measurement, dried metabolite extracts were derivatized using an automated derivatization robot (Gerstel MPS). The first derivatization was performed by adding 15 µL of (20 mg/mL) methoxyamine hydrochloride in pyridine (Sigma-Aldrich), shaken for 90 min at 40 °C. The second derivatization was performed by adding an equal volume of N-methyl-N-trimethylsilyl-trifluoroacetamide (MSTFA) (Macherey-Nagel) under continuous shaking for 30 min at 40 °C. The sample (1 µL) was injected into an SSL injector at 270 °C in spitless mode. GC-MS analysis was performed using an Agilent 7890A GC equipped with a 30 m DB-35MS + 5 m Duraguard capillary column (0.25 mm inner diameter, 0.25 µm film thickness). Helium was used as the carrier gas at a flow rate of 1.0 mL/min. The GC oven temperature was held at 80 °C for 6 min, subsequently increased to 300 °C at 6 °C/min, and held at that temperature for 10 min. The temperature was increased to 325 °C at 10 °C/min and held for an additional 4 min, resulting in a total run time of 60 min per sample. The GC was connected to an Agilent 5977B MSD. The transfer line temperature was set to 280 °C, and the MSD was operating under electron ionization at 70 eV. The MS source was held at 230 °C and the quadrupole at 150 °C. Full scan mass spectra were acquired from m/z 70 to m/z 800 at a scan rate of 5.2 scans/s. Pooled samples were measured after every eighth GC-MS measurement for quality control and data correction [35].

### 2.5. Data Processing

Deconvolution of mass spectra, peak picking, integration, and retention index calibration was performed using our in-house software [36]. Compounds were identified using an in-house mass spectral library by spectral and retention index similarity. The following deconvolution settings were applied to scan data: peak threshold: 5; minimum peak height: 5; bins per scan: 10; deconvolution width: 7; no baseline adjustment; minimum 15 peaks per spectrum; no minimum required base peak intensity. Retention index calibration was based on a C10–C40 even n-alkane mixture (68281, Sigma-Aldrich, Munich, Germany). Relative quantification was carried out using the batch quantification function of our in-house software [36]. Data were normalized to quality control pool measurement and intensity of the internal standard (D6-Glutaric acid).

### 2.6. Statistical Analysis

Metabolomics data were further processed using Metaboanalyst 5.0 [37]. Cube root transformation and range scaling methods were applied to obtain Gaussian distribution. Principal component analysis (PCA) was performed to identify intrinsic clustering. Supervised clustering was performed using partial least squares discriminant analysis (PLS-DA). Further, the model accuracy was tested using cross-validation by 100 permutations to avoid over-fitting. R2 and Q2 values were used to assess the goodness of the fit and predictive ability of the PLS-DA model. The groups’ (COVID-19 disease, non-COVID-19 pneumonia, and controls) significant metabolic differences were identified using ANOVA (*p* < 0.05) adjusted for multiple hypothesis testing using FDR correction. A post-hoc analysis using Tukey’s HSD was performed to identify within groups’ (COVID-19 disease against controls and non-COVID-19 pneumonia against controls) significance. Arterial and venous sample differences from the same individuals were identified by performing repeated measures ANOVA in r [38]. Box-and-whisker plots and ROC curve analysis plots were plotted using r packages. Heat maps of differentially expressed metabolites were created using Metaboanaylst 5.0. Significant metabolites were further submitted for pathway analysis using a pathway analysis tool (MetPA) in Metaboanalyst 5.0 [37].

To analyze the transpulmonary gradients of cytokine concentrations, we determined the difference between the concentrations obtained from samples from the peripheral arterial catheter and the central venous catheter (CVC) (Δ-concentration). These Δ-concentrations were compared between the three patient groups (ANOVA test). Furthermore, the significant difference between the venous and arterial concentration of each cytokine within each group was analyzed with paired *t*-test. A significance value of *p* ≤ 0.05 was applied to all tests, and SPSS V27 (IMB) was used for analysis.

### 2.7. Machine Learning Approaches

Machine learning approaches such as support vector machines (SVM) were employed to identify the predictive marker metabolites for disease outcomes. In brief, the synthetic minority oversampling technique (SMOTE) was used to fit imbalances for the training set [39]. SMOTE takes the minority class data points and creates new data points which lie between any two nearest data points joined by a straight line. Hyperparameter tuning was performed on the SVM classifier with training data. SVM-recursive feature elimination (RFE) was used to select features (metabolites) for the classification algorithm [40]. Essentially, this method trains the model on the original number of features, and importance is given to each feature. The least important features are taken out, and then the process is repeated to a specified number of features. Finally, cross-validation was used to determine an optimal number of features. For each iteration of splitting the data into test and hold-out set, different features were selected by SVM-RFE. Only those features selected by SVM-RFE in each iteration were used for the training of the SVM classifier. The SVM classifier was trained on the 10 train datasets to evaluate variability handling with the classifier.

## 3. Results

### 3.1. Exploratory Statistical Analysis of Plasma Metabolites

**Figure 1:**
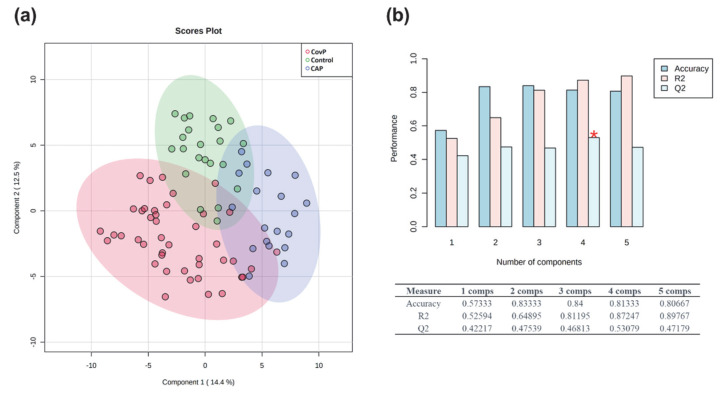
Exploratory multivariate statistical analysis. (**a**) Partial least square discriminant analysis (PLS-DA) score plot depicting clustering of COVID-19 pneumonia (CovP), control, and non-COVID-19 pneumonia (CAP) samples. (**b**) Plot obtained after performing a random permutation test with 100 permutations on PLS-DA model. The red asterisk indicates the best classifier (R2 = 0.87, Q2 = 0.53), R2 is the explained variance, and Q2 is the predictive ability of the model. Q2 represents the model’s predictive ability and is calculated by comparing the predicted data with the original data. The calculated prediction error (Predicted Residual Sum of Squares or PRESS) is divided by the initial sum of squares and subtracted from 1. High R2 and Q2 values represent the model’s good predictive ability and confirm our PLS-DA model’s validity. The inset table summarizes Q2, R2, and the accuracy of the best model. Comps mean the number of components.

We performed metabolic profiling of plasma samples collected from 43 COVID-19 pneumonia, 23 non-COVID-19 pneumonia, and 26 control subjects. Data processing using our in-house software resulted in the detection of 157 metabolites detectable across all samples [36], of which the structural identity of 67 metabolites was confirmed by using an in-house metabolic reference library. Metabolite levels were normalized to the internal standard and pooled quality control samples. In addition, the data matrix was log-transformed and Pareto scaled for further statistical analysis. Principal component analysis (PCA) revealed inherent clusters among the sample groups of COVID-19 pneumonia, non-COVID-19 pneumonia, and controls based on the metabolic profile (Figure S1). Moreover, a partial least square discriminant analysis (PLS-DA) revealed clear discrimination among the three groups (Figure 1a). A cross-validation analysis using 100 randomly permutated models indicated a good predictive ability of the original PLS-DA model with cumulative R2 and Q2 values of the model 0.87 and 0.53, respectively (Figure 1b). Q2 represents the predictive ability of the model and is calculated by comparing the predicted data with the original data.

### 3.2. Significant Metabolic Alterations

**Figure 2:**
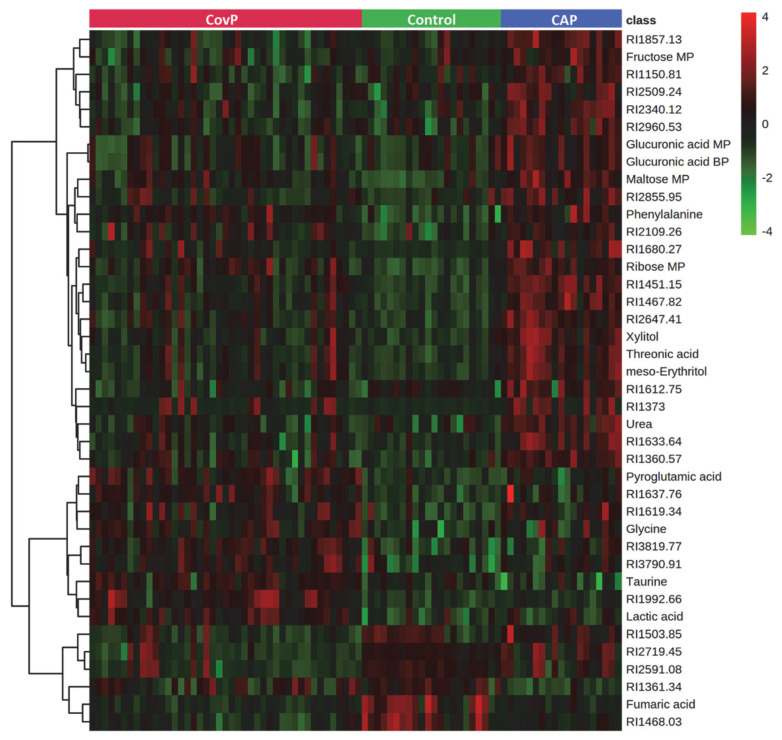
Heatmap of top 40 significantly altered metabolites in group comparison (COVID-19 pneumonia (CovP), control subjects, and non-COVID-19 pneumonia (CAP)) selected after ANOVA (*p* < 0.05). The colors from green to red indicate the increased concentration (normalized peak area) of metabolites.

Metabolic alterations among the groups were identified using ANOVA (*p*-value < 0.05), adjusted for multiple hypotheses testing by false discovery rate (FDR). Metabolites, discriminating all three groups, were selected using post-hoc analysis. Overall, 66 metabolites showed significant alterations among COVID-19 pneumonia, non-COVID-19 pneumonia, and controls (Supplementary Table S3). These include amino acids, fatty acids, amino sugar derivatives, and organic acids. Unknown metabolites that were not matched with the in-house library are annotated using their retention indices. An overview of the top 40 differentially altered metabolites among COVID-19 pneumonia, non-COVID-19 pneumonia, and controls are depicted in Figure 2. Further post-hoc analysis (Tukey’s HSD) uncovered the specific differences between the groups (Supplementary Table S3). Significant differences in the levels of 29 metabolites were observed in COVID-19 pneumonia compared to the control subjects. Levels of aspartic acid, galactose, glycine, lactic acid, lyxose, maltose, ornithine, phenylalanine, pyroglutamic acid, ribose, serine, and taurine were increased while fumaric acid levels were decreased in COVID-19 pneumonia patients (Figure S2). In the non-COVID-19 pneumonia compared to the control subjects’ group comparison, 37 metabolites were identified as significant. Wherein, we observed increased levels of erythrose 4-phosphate, fructose, gluconolactone, gluconic acid, glucuronic acid, isoleucine, meso-erythritol, methionine, threonic acid, tyrosine, urea, and xylitol in non-COVID-19 pneumonia patients (Figure S3).

### 3.3. Pathway Analysis

**Figure 3:**
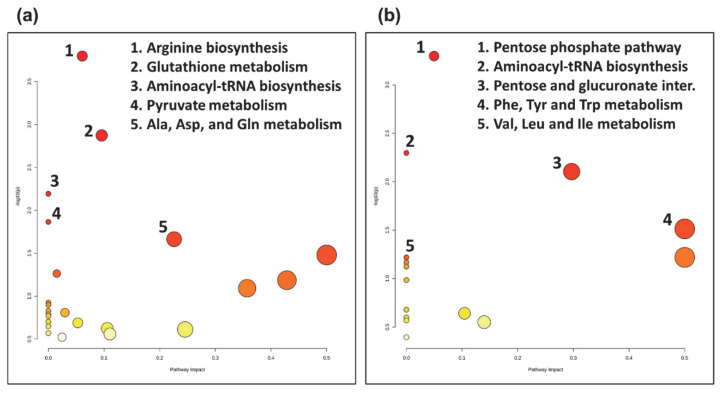
Topology map of altered metabolic pathways describing the impact of metabolites selected from comparative post-hoc analysis (Tukey’s HSD). (**a**) The top five altered metabolic pathways in the COVID-19 pneumonia (CovP) group. 1. Arginine biosynthesis, 2. Glutathione metabolism, 3. Aminoacyl-tRNA biosynthesis, 4. Pyruvate metabolism, 5. Alanine, aspartate, and glutamate metabolism. (**b**) The top five altered metabolic pathways in the non-COVID-19 pneumonia (CAP) group. 1. Pentose phosphate pathway, 2. Aminoacyl-tRNA biosynthesis, 3. Pentose and glucuronate interconversions, 4. Phenylalanine, tyrosine, and tryptophan metabolism, 5. Valine, leucine and isoleucine metabolism.

Metabolites selected as significant from the post-hoc analysis between the two groups, COVID-19 pneumonia and non-COVID-19 pneumonia, were further subjected to pathway analysis. Interestingly, pathway analysis revealed distinct metabolic responses in the COVID-19 pneumonia and non-COVID-19 pneumonia group. The top five pathways that were enriched in the COVID-19 pneumonia group were arginine biosynthesis, glutathione metabolism, aminoacyl-tRNA biosynthesis, pyruvate metabolism, and alanine, aspartate, and glutamate metabolism (Figure 3a). In comparison, the top five pathways altered in the non-COVID-19 pneumonia group were the pentose phosphate pathway, aminoacyl-tRNA biosynthesis, pentose and glucuronate interconversions, phenylalanine, tyrosine and tryptophan metabolism, and valine, leucine and isoleucine metabolism (Figure 3b).

### 3.4. Machine Learning Signature to Predict Disease Outcome

**Figure 4:**
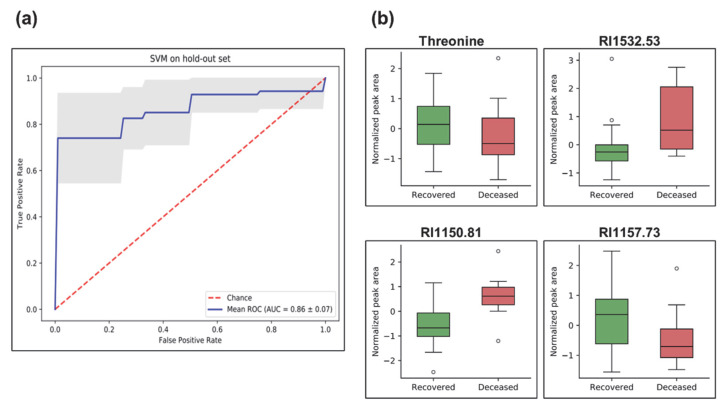
Machine learning analysis using support vector machine (SVM) depicting prediction of disease outcome (recovered vs. deceased) in COVID-19 pneumonia subjects. (**a**) Receiver operating characteristic (ROC) plot of the true positive rate (i.e., sensitivity) and the false positive rate (i.e., 1-specificity). ROC is used to evaluate classification models that classify subjects into one of two categories (recovered or deceased). The area under the ROC curve provides a way to measure the accuracy (1 highest and less than 0.5 low). The present classifier correctly classifies the cohort for disease outcome (recovered vs. deceased) with an area under (AUC) the ROC of 86 ± 10%. The blue line is the mean and the grey area is the standard deviation of all ROCs of train split. (**b**) Box-and-whisker plots of metabolites used in the SVM classifier model, illustrated as normalized peak area differences between recovered (green box) vs. deceased (red box) subjects.

Next, we applied a machine learning approach based on a support vector machine (SVM) to disclose the metabolic signature that can predict the COVID-19 outcome (recovered vs. deceased). Among the 43 COVID-19 patients recruited for this study, 32 recovered and 10 deceased. At first, we applied the synthetic minority oversampling technique (SMOTE) to fit imbalances in the training dataset. Hyperparameter tuning was performed using repeated randomized cross-validation on the SVM classifier with training data. Subsequently, SVM-recursive feature elimination (RFE) was applied to select metabolite features for the classification algorithm. RFE aims to select features by recursively considering smaller and smaller sets of features. Finally, cross-validation was employed to determine an optimal number of features. The SVM classifier was trained on the 10 training datasets to evaluate variability handling with the classifier. We applied receiver operating characteristic (ROC) to evaluate our classification model. The classifier correctly predicted outcomes (recovered vs. deceased) of the COVID-19 subjects with an area under the ROC curve (AUC) of 86 ± 10% (Figure 4a). Box-whisker plots of the concluding four marker metabolites (threonine, RI1532.53, RI1557.73, and RI1150.81) predictive of disease fatality (recovered vs. deceased) in COVID-19 individuals are presented in Figure 4b. In addition, the predictive power of the final model was evaluated via a precision–recall curve. Precision is the ratio of the number of true positives divided by the sum of true positives and false positives, which characterizes the ability of the model to predict the positive samples correctly. It is complemented by the recall metric, which is the ratio of true positives to the actual positives of the data. A perfect model will have a precision and recall of 1 for every chosen threshold. The information of the precision–recall curve is summarized via the AUC metric (Figure S4).

### 3.5. Effect of Pneumonia on the Plasma Metabolome and Cytokines

**Figure 5:**
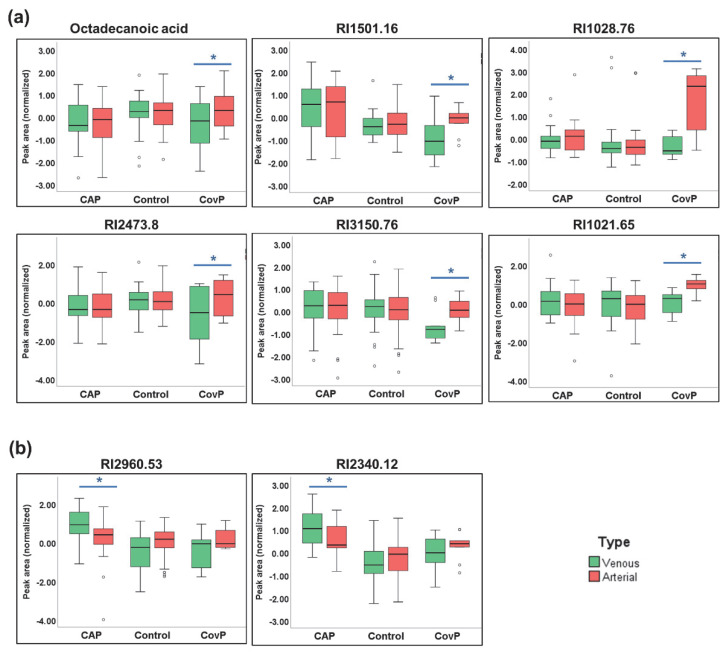
Significant metabolite differences in venous and arterial samples revealed by repeated-measures ANOVA. (**a**) Box-and-whisker plots of COVID-19 pneumonia (CovP) specific significant metabolic differences in venous (green box) and arterial samples (red box). (**b**) Box-and-whisker plots of non-COVID-19 pneumonia (CAP) specific significant metabolic differences in venous (green box) and arterial samples (red box). (Asterisk indicates *p* ≤ 0.05).

We then set out to elucidate the plasma metabolic effects of pneumonia specific for COVID-19 and non-COVID-19 pneumonia. Accordingly, we compared the plasma metabolome of systemic venous (pre-lung) and arterial (post-lung) blood samples from the same individuals. To identify significant metabolic differences in arterial vs. venous samples, we performed a repeated-measures ANOVA. As a result, significant differences in nine metabolite levels were revealed across arterial and venous samples specific for COVID-19 pneumonia (Figure 5a). We observed increased levels of octadecanoic acid, RI1501.16, RI1028.76, RI2473.8, RI3150.76, RI1021.65, RI3150.76, and RI1021.65 in the arterial samples, reflecting the contribution from lung metabolism. Contrarily, in arterial vs. venous samples from non-COVID-19 pneumonia patients’ levels of two metabolites (RI2960.53 and RI2350.12) were significantly lower (Figure 5b).

**Figure 6:**
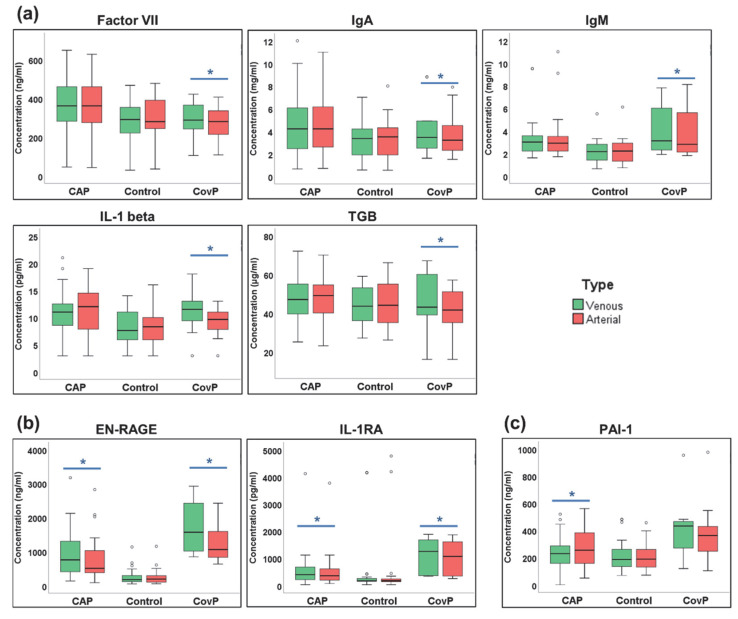
Significant cytokines differences in venous and arterial samples. (**a**) Box-and-whisker plots of COVID-19 pneumonia (CovP) specific significant cytokine differences. (**b**) Box-and-whisker plots of both non-COVID-19 pneumonia (CAP) and COVID-19 specific significant cytokine differences; (**c**) Box-and-whisker plots of both non-COVID-19 pneumonia (CAP) specific significant cytokine differences. (Venous (green box), arterial samples (red box), an asterisk indicates *p*-value ≤ 0.05).

Next, we investigated whether the blood concentrations of cytokines were changed after the passage of the pulmonary circulation (Supplementary Table S4). A paired *t*-test was employed to compare venous (pre-lung) and arterial (post-lung) samples. Significant differences in Factor VII, IgA, IgM, IL-1beta, and TGB were observed in the COVID-19 group (Figure 6a), while EN-RAGE and IL-1RA showed significant alteration in both COVID-19 and non-COVID-19 pneumonia groups (Figure 6b). PAI-1 levels were significant in the non-COVID-19 pneumonia group (Figure 6c).

**Figure 7:**
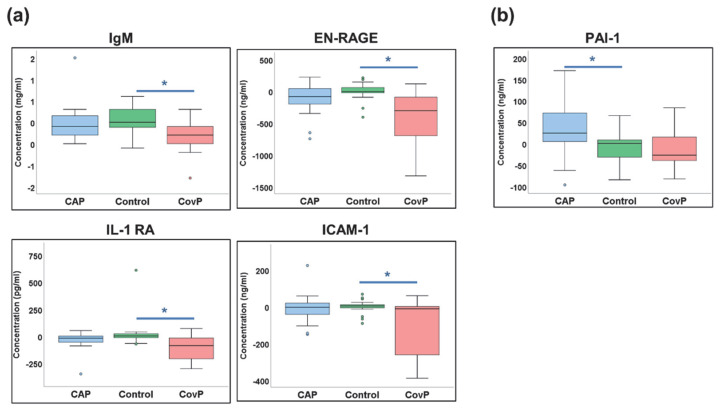
Cytokines with significant differences in the delta values. (**a**) Box-and-whisker plots of COVID-19 pneumonia (CovP) specific significant differences in delta values as compared to controls. (**b**) Box-and-whisker plots of both non-COVID-19 pneumonia (CAP) specific significant cytokine differences in delta values as compared to controls. [non-COVID-19 pneumonia (blue box), Control samples (green box), COVID-19 pneumonia (red box), an asterisk indicates *p*-value ≤ 0.05].

Next, we compared the differences between venous and arterial blood samples (“Δ-concentrations”) between the groups. Here, cytokines IgM, EN-RAGE, IL1-RA, and ICAM-1 showed significantly different Δ-concentrations between COVID-19 pneumonia and the control group (Figure 7a). While cytokine PAI-1showed significant differences in Δ-concentrations between non-COVID-19 pneumonia and control groups (Figure 7b). Overall, these data revealed that the composition of the blood metabolome and cytokine pattern within the lung is disease-specific and differs between COVID-19 and non-COVID-19 pneumonia.

## 4. Discussion

In this study, we employed a non-targeted plasma metabolomics approach to identify group-specific differences among COVID-19 pneumonia, non-COVID-19 pneumonia, and control groups (Supplementary Table S3). Disease-specific metabolic alterations were detected in COVID-19 pneumonia and non-COVID-19 pneumonia groups (compared to controls unless otherwise specified) and can be used to develop biomarker patterns predictive of disease fatality. A transpulmonary gradient of metabolites and cytokines was found for several metabolites and cytokines highlighting the role of the lung in the modulation of systemic disease consequences.

Plasma concentrations of amino acids were specifically altered in the COVID-19 pneumonia group such as aspartic acid, glycine, and serine (Figure S2). An increase in the levels of glycine has previously been linked with COVID-19 infection [41]; an increase in glycine activates porphyrin metabolism, which is a key step for disease progression [41]. Notably, taurine levels were specifically increased in the COVID-19 pneumonia group while these levels were slightly decreased in the non-COVID-19 pneumonia group. In humans, leucocytes have been described to have the highest levels of taurine [42], where it acts as an antioxidant affecting immune function [43]. Alternately, taurine metabolism has also been shown to influence sepsis by altering the release of important inflammatory mediators [44], indicating a similar response in COVID-19 pneumonia. In line with this, lactic acid levels were significantly increased in the COVID-19 pneumonia group. Lactic acid has been shown to affect immune functions via the regulation of immune cell-specific signaling pathways [45,46]; lactic acidosis contributes to the inflammatory response through dysregulation of cytokines and macrophage activation [47]. Lactic acid is also strongly associated with sepsis, which can be attributed to mitochondrial dysfunction [48,49,50]. Thus, increased lactic acid levels in the plasma of COVID-19 pneumonia patients are indicative of a sepsis-induced inflammatory response [51].

Significant changes in the levels of the essential amino acids viz. methionine, tyrosine, and isoleucine were observed in the non-COVID-19 pneumonia group (Figure S3). An increase in the levels of methionine and tyrosine could be related to bacterial infection as these are amino acids that are typical for pathogen metabolism [52]. Isoleucine, a branched-chain amino acid, acts as a transcriptional regulator in bacterial pathogenesis [53]. It functions as a host metabolic signal that regulates virulence gene expression in certain bacteria, including a common pneumonia-causing bacteria *Staphylococcus aureus* [53]. We also observed higher urea levels in non-COVID-19 pneumonia individuals, which corroborates with previous reports [54]. In non-COVID-19 pneumonia subjects, pneumonia is often followed by dehydration which results in increased reabsorption of urea by the kidney. Earlier studies have also reported an association of urea levels with mortality and the severity of non-COVID-19 pneumonia [55].

Interestingly, our pathway analysis revealed distinct metabolic responses in COVID-19 pneumonia patients (Figure 3a). Arginine metabolism plays a vital role in the immune response [56]. Arginine is the direct precursor in nitric oxide (NO) synthesis, a key signaling molecule [57]. Altered arginine metabolism is also indicative of the oxidative response and can contribute to endothelial dysfunction observed in COVID-19 pneumonia [58]. Altered arginine metabolism has also been reported in previous COVID-19 studies [59,60]. Glutathione metabolism is extremely important for the regulation of cellular ROS and the function of the immune system [61]. Moreover, it has been shown that COVID-19 pneumonia affects redox cellular homeostasis, a key step in the cytopathic effects of viral infections [62]. Notably, viral infections including COVID-19 induce increased glycolytic flux along with an increased pyruvate metabolism [63,64]. Viral infections primarily target glycolysis by modulating glucose transporters, which are important for the host cell response and immune activation [65]. Moreover, alterations in alanine-, aspartate-, and glutamate- metabolism were also observed in COVID-19 pneumonia patients. This pathway is essential in the generation of GABA, a signaling molecule with omnipresent receptors on various immune cells [66]. GABA has been shown to modulate inflammation and clearance of alveolar fluid in acute lung injury [67].

In comparison, impacted pathways in non-COVID-19 pneumonia patients revealed different metabolic responses (Figure 3b). The pentose phosphate pathway is important for polyamine metabolism and suggestive of an oxidative stress response to infection due to its NADPH supply [68]. Aminoacyl-tRNA synthesis is vital for protein synthesis and is indicative of response to infection by regulating transcription, translation, and various signaling pathways [69]. Pentose and glucuronate interconversions pathways lay a pivotal role in the clearance of toxic substances [70]. Toxic substances are cleared by conjugation with other compounds to mask the toxic groups. D-glucuronic acid is a key molecule that binds to toxic substances assisting their clearance through the pentose and glucuronate interconversions pathway, which is increased in the non-COVID-19 pneumonia group [70]. Phenylalanine, tyrosine, and tryptophan biosynthesis could be explained by the catabolism of muscle protein as a response to infection; leading to the release of phenylalanine, which is used for the synthesis of inflammatory molecules [71]. Valine, leucine, and isoleucine (BCAA) metabolism stipulate aggressive glucose consumption and subsequent amino acid synthesis, which corroborates with earlier reports as well [72].

In addition, we identified a panel of four metabolites that could predict the outcome (recovered vs. deceased) of the COVID-19 individuals. Among three unknown metabolites, the structural identity of threonine was confirmed by our in-house library. This particular amino acid has been shown to be one of the direct indicators of inflammatory diseases such as sepsis [73]. As demonstrated in rats, a decrease in threonine levels was directly correlated with an increased synthesis of both mucins and gut epithelial proteins during sepsis [73]. The reduced levels of threonine in the plasma of deceased COVID-19 pneumonia subjects could be indicative of the severity of sepsis and may serve as a good marker to predict the disease outcome [74]. Moreover, the strong correlation of these four metabolites in predicting disease outcomes for COVID-19 warrants future targeted investigation.

Since COVID-19 pneumonia is a heterogeneous disease distinguished by thrombosis, pulmonary embolism, and inflammatory cell infiltration [75,76], we expected strong modulations of plasma metabolites and inflammatory cytokines in the lung. Pinpointing differences in the blood composition of the lung artery and vein could provide a snapshot of biochemical and inflammatory activities happening during pneumonia. We revealed significant concentration changes between venous and arterial blood in nine metabolites in COVID-19 pneumonia and in two metabolites in non-COVID-19 pneumonia (Figure 5). Interestingly, the fatty acid octadecanoic acid (stearic acid) was significantly altered in the venous and arterial comparison of the COVID-19 pneumonia group. Octadecanoic acid has previously been reported to be altered in bronchoalveolar lavage fluid of COVID-19 subjects [77]. Octadecanoic acid has been shown to decrease nuclear factor-kB activity and inflammatory cell accumulation [78]. Octadecanoic acid may exert some regulatory effect on fibrogenesis by suppressing myofibroblast differentiation [79]. Additionally, the accumulation of saturated fatty acids such as octadecanoic acid is an indicator of disruption of the structural and functional characteristics of the cells inducing cell death mediated by apoptosis or necrosis [80]. Other metabolic entities identified as transpulmonary activities need further structural confirmation, concerning their role in lung pathogenesis of COVID-19 pneumonia and non-COVID-19 pneumonia. Several cytokines have significant transpulmonary gradients within non-COVID-19 pneumonia and COVID-19 disease, more prominent in the COVID-19 group. As expected, no differences were observed within the control group. The venous concentration of cytokines correlated with disease severity and outcome in an earlier study [34].

Overall, our study provides unique insights into the pathophysiology of pneumonia in COVID-19 and non-COVID-19 pneumonia using metabolic differences. The study has limitations and strengths. The patient sample includes more men than women, which might cause a bias. We initially included only male patients to account for cyclic changes of the blood metabolome in female individuals. With the beginning of the COVID-19-pandemics, we decided to also include female patients to address this novel disease. Moreover, we did not correct for several underlying factors such as diet or medication. The strength of the study is based on the unique patient collection that allowed for various biosamples to study the transpulmonary gradient of cytokines and metabolites. Threonine along with three other metabolites could serve as predictive biomarkers for COVID-19 disease severity. We observed systematic changes in transpulmonary levels of octadecanoic acid along with six other metabolites in COVID-19 subjects that could be indicative of fibrogenesis. Similarly, prominent changes in transpulmonary levels of cytokines were also observed in COVID-19 subjects. These data highlight the role of the lung in the modulation of systemic inflammation and might also help to understand how lung infection caused dysfunction in other organs.

## Figures and Tables

**Table 1:** Distribution of age, sex, and BMI between the study groups.

Item	Control	Non-COVID-19 Pneumonia	COVID-19 Pneumonia
Patient number	26	23	43
Sex, % male	100	100	65
Age	62.54 (11.39)	65.57 (14.88)	55.98 (27.44)
BMI	26.37 (5.34)	27.27 (9.67)	27.22 (6.07)

Data are mean (SD) and n (%).

## Data Availability

Not applicable.

## Supplementary Materials

The following supporting information can be downloaded at: https://www.mdpi.com/article/10.3390/metabo12111058/s1, Figure S1: Principal component analysis score plot depicting clustering of COVID-19 pneumonia (CovP) (red), control subjects (Controls) (Green), and non-COVID-19 pneumonia (CAP) (Blue); Figure S2: Box-and-whisker plots of COVID-19 pneumonia (CovP) specific significant metabolic differences as compared to controls obtained after Tukey’s HSD and illustrated as normalized peak area differences; Figure S3: Box-and-whisker plots of non-COVID-19 pneumonia (CAP) specific significant metabolic differences as compared to controls obtained after Tukey’s HSD and illustrated as normalized peak area differences; Figure S4: Precision recall curve; Table S1: Summary of the comorbidities of the study cohort; Table S2: Detailed characteristics of all COVID-19 patients; Table S3: Significant metabolic differences among COVID-19 pneumonia (CovP), Control subjects, and non-COVID-19 pneumonia (CAP) revealed after ANOVA; Table S4: List of all cytokines with their means of delta values, standard deviation and median.
