# Supplementary material for: Plasma Metabolome Alterations Discriminate between COVID-19 and Non-COVID-19 Pneumonia
Source: Metabolites. 2022 Nov 2;12(11):1058. doi: 10.3390/metabo12111058 (PMC9693035; doi:10.3390/metabo12111058)
Supplement: Supplementary file 1 [file metabolites-12-01058-s001.zip › metabolites-1928069-supplementary.pdf]

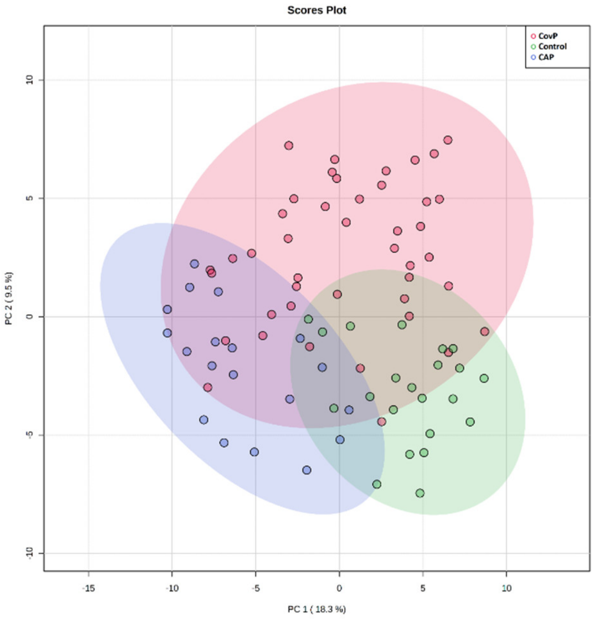

Figure S1. Principal component analysis score plot depicting clustering of COVID-19 pneumonia (CovP) (red), control subjects (Controls) (Green), and non-COVID-19 pneumonia (CAP) (Blue).

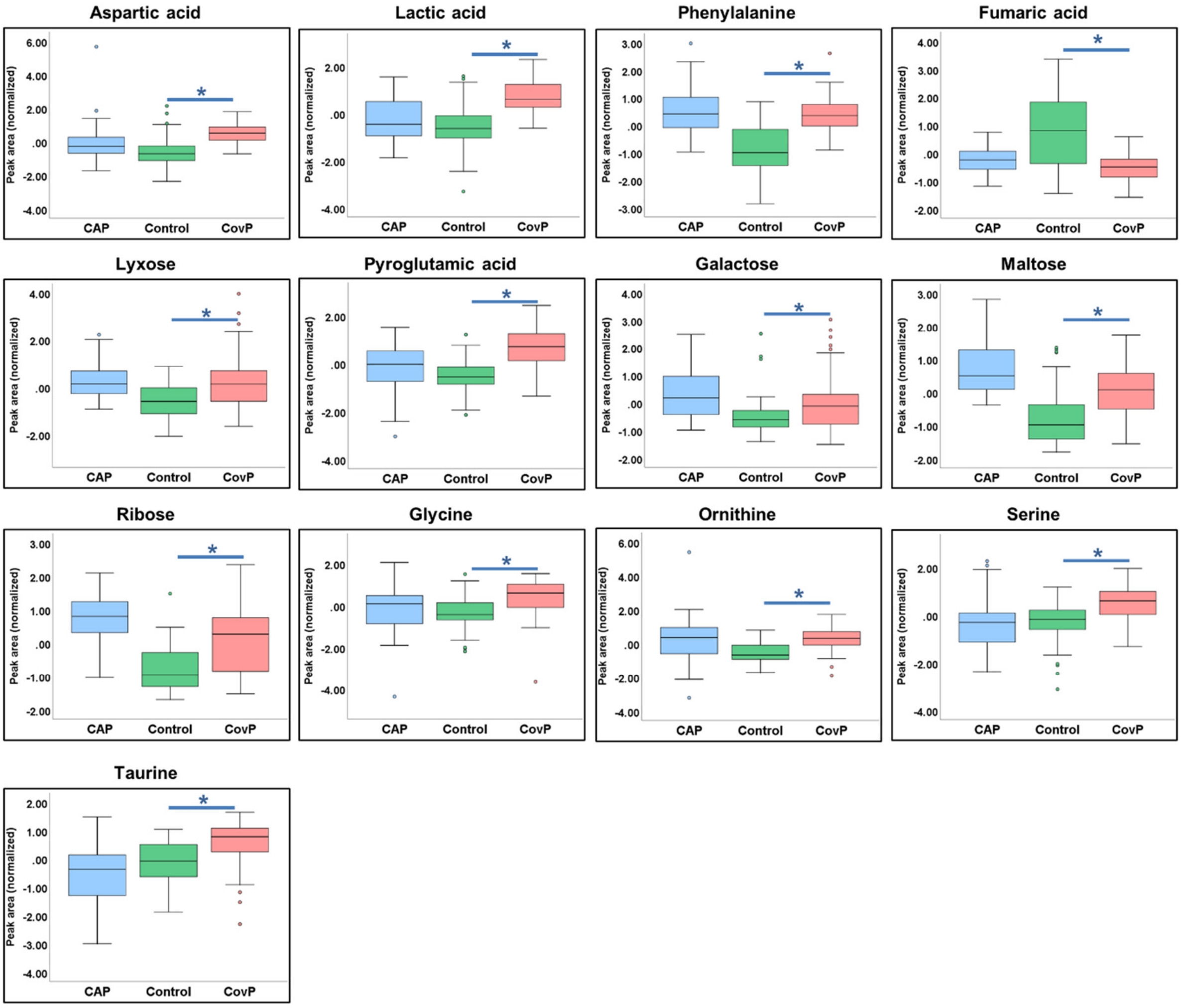

Figure S2. Box-and-whisker plots of COVID-19 pneumonia (CovP) specific significant metabolic differences as compared to controls obtained after Tukey's HSD and illustrated as normalized peak

area differences. [Non-COVID-19 pneumonia (blue box), Control samples (green box), COVID-19 pneumonia (red box), an asterisk indicates p-value ≤ 0.05].

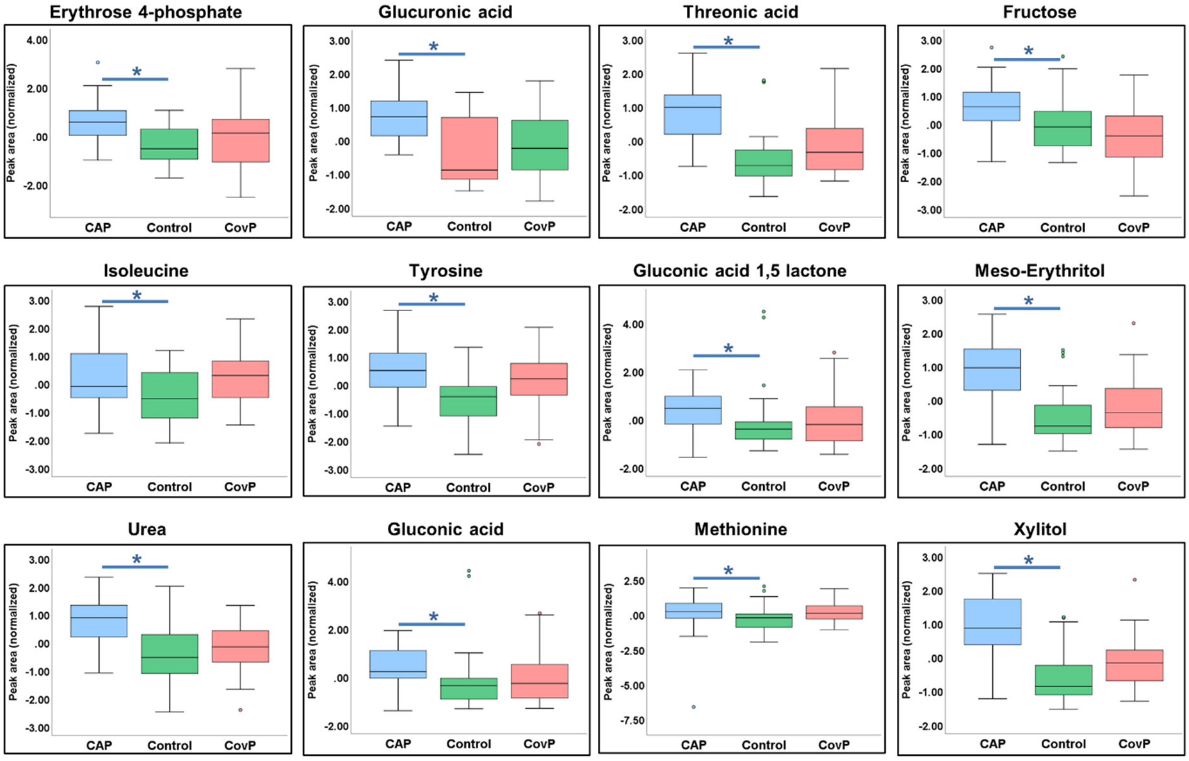

Figure S3. Box-and-whisker plots of non-COVID-19 pneumonia (CAP) specific significant metabolic differences as compared to controls obtained after Tukey's HSD and illustrated as normalized peak area differences. [non-COVID-19 pneumonia (blue box), Control samples (green box), COVID-19 pneumonia (red box), an asterisk indicates p-value ≤ 0.05].

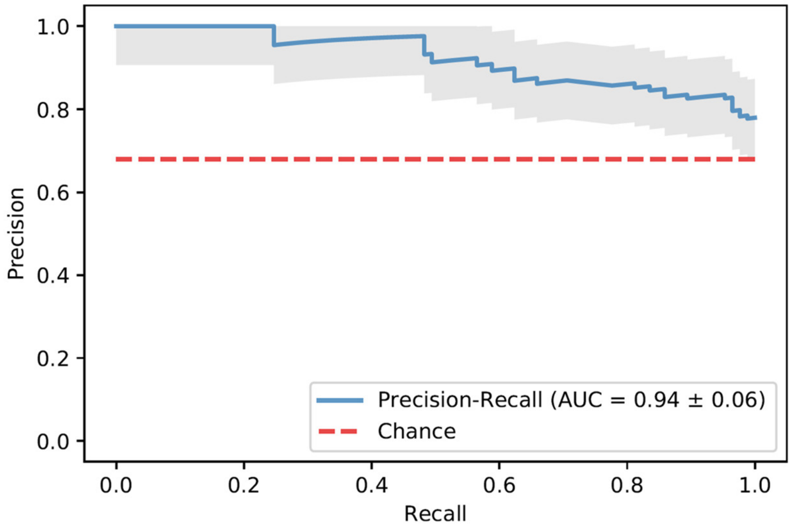

Figure S4. Precision recall curve. Precision is the ratio of the number of true positives divided by the sum of true positives and false positives, which characterizes the ability of the model to predict the positive samples correctly. It is complemented by the recall metric, which is the ratio of true positives to the actual positives of the data. A perfect model will have a precision and recall of 1 for every chosen threshold.

Table S1. Summary of the comorbidities of the study cohort.

Comorbidity	Control	Non-COVID-19 Pneumonia	COVID-19 Pneumonia
Arterial hypertension	14 (53.8%)	15 (65.2%)	9 (20.9%)
Myocardial infarction	2 (7.7%)	7 (30.4%)	0 (0%)
Cerebrovascular accident	2 (7.7%)	6 (26.1%)	0 (0%)
Vein thrombosis	0 (0%)	0 (0%)	0 (0%)
Liver cirrhosis	2 (7.7%)	1 (4.3%)	0 (0%)
Hyperthyroidism	2 (7.7%)	0 (0%)	2 (4.7%)
Hyporthyroidism	0 (0%)	2 (8.7%)	0 (0%)
Diabetes type without Insulin	8 (30.8%)	3 (13%)	3 (7%)
Diabetes type 2 with Insulin	0 (0%)	6 (26.1%)	0 (0%)
Hyperlipidemia	2 (7.7%)	6 (26.1%)	1 (2.3%)
Anemia	4 (15.4%)	5 (21.7%)	0 (0%)
Cancer	20 (76.9%)	2 (8.7%)	5 (11.6%)
Osteoporosis	1 (3.8%)	0 (0%)	0 (0%)
Smoking	0 (0%)	7 (35%)	0 (0%)
Ischemic brain insult	0 (0%)	0 (0%)	3 (7%)
Aallergies	0 (0%)	0 (0%)	3 (7%)
Hepatitis	0 (0%)	0 (0%)	1 (2.3%)
Kidney transplant	0 (0%)	0 (0%)	1 (2.3%)
Asthma	0 (0%)	0 (0%)	2 (4.7%)
Cholelithiasis	0 (0%)	0 (0%)	2 (4.7%)
Chronic Kidney disease	0 (0%)	0 (0%)	2 (4.7%)
COPD	0 (0%)	0 (0%)	1 (2.3%)
Coronary artery disease	0 (0%)	0 (0%)	1 (2.3%)
Depression	0 (0%)	0 (0%)	1 (2.3%)
Dilative cardiomyopathy	0 (0%)	0 (0%)	1 (2.3%)
Epilepsy	0 (0%)	0 (0%)	1 (2.3%)
Diverticulosis	0 (0%)	0 (0%)	1 (2.3%)
Parkinson	0 (0%)	0 (0%)	1 (2.3%)
Myeloplast. syndrom	0 (0%)	0 (0%)	1 (2.3%)
Nephrolithiasis	0 (0%)	0 (0%)	1 (2.3%)
Plasmozytoma	0 (0%)	0 (0%)	1 (2.3%)
Pulmonary artery embolisms	0 (0%)	0 (0%)	1 (2.3%)
Autoimmune disease	0 (0%)	0 (0%)	1 (2.3%)

Table S2. Detailed characteristics of all COVID-19 patients. [Disease severity, 1 = ormal care unit, 2 = intensive care unit; NA = not available].

#	Age	Sex	Disease severity	Death	Height (cm)	Weight (kg)	Comorbidity
1	3	F	1	No	100	15	-
2	7	M	1	No	135	30	-
3	72	M	1	No	177	86	Reflux esophagitis, colon diverticulosis, NHL
4	12	M	1	No	142	30	-
5	17	M	1	No	0	87	-
6	66	F	NA	No	NA	NA	-
7	85	F	1	No	NA	NA	-
8	15	F	1	No	169	56	-
9	5	M	1	No	125	23	-
10	91	M	1	No	NA	NA	-
11	5	M	1	No	NA	NA	-
12	52	M	2	No	186	93	-
13	64	M	2	No	NA	NA	-
14	50	M	2	No	NA	NA	Hyperthyroidism

15	51	M	1	No	NA	NA	M. Parkinson
16	58	M	1	No	175	100	Art. Hypertension
17	54	F	1	No	175	55	Pulmonary artery embolisms, autoimmune disease
18	23	F	1	No	169	75	Epilepsy, allergies
19	61	F	1	No	163	92	Breast cancer, art. Hypertension
20	52	F	1	No	169	89	Dilative cardiomyopathy, Diabetes mell. Type 2, Asthma bronchiale
21	69	M	1	No	182	84	Chron. Kidney disease, coronary arterial disease, Diabetes mell. type 2
22	40	F	1	No	NA	NA	-
23	76	M	1	No	173	70	-
24	80	F	1	No	164	66	Hyperlipidemia, art. hypertension, breast cancer, diverticulosis
25	28	F	1	No	158	45	Asthma bronchiale
26	79	M	1	No	175	100	Art. Hypertension
27	88	M	1	No	165	45	-
28	67	M	1	No	184	114	Art. Hypertension, Hepatitis
29	90	F	1	No	NA	NA	Art. Hypertension, Diabetes mell. Type 2, ischemic brain insult, breast cancer
30	85	M	1	No	NA	NA	Chronic kidney disease
31	82	M	1	No	NA	NA	ischemic brain insult, hyperthyroidism
32	89	F	1	No	NA	NA	-
33	13	M	1	No	NA	44	Depression
34	59	M	1	Yes	182	80	Art. Hypertension, Diabetes mell. type 2, allergies
35	55	M	2	Yes	NA	NA	Nephrolithiasis, cholelithiasis
36	49	M	2	Yes	185	120	Cholelithiasis
37	86	M	2	Yes	NA	NA	Coronary artery disease
38	70	W	1	Yes	NA	66.5	Art. hypertension, kidney transplant
39	71	F	2	Yes	182	88	Art. hypertension, allergies
40	60	M	1	Yes	NA	NA	ischemic brain insult
41	85	F	1	Yes	1.7	73	Myeloplast. syndrom
42	81	M	2	Yes	172	89	Plasmozytoma, prostate cancer-Ca
43	62	M	1	Yes	170	108	COPD, lung cancer

Table S3. Significant metabolic differences among COVID-19 pneumonia (CovP), Control subjects, and non-COVID-19 pneumonia (CAP) revealed after ANOVA. [Table sorted according to post hoc analysis; p-value: p-values obtained after performing ANOVA (p-value<0.05); FDR: value obtained after performing false discovery test; Tukey's HSD: result depicting group-specific significance after post-hoc analysis (Tukey's HSD); CovP/control: metabolic concentration (peak area) differences in COVID-19 pneumonia (CovP) compared to control subjects (↑ increased, ↓ decreased); CAP/control: metabolic concentration differences (peak area) in non-COVID-19 pneumonia compared to control subjects (↑ increased, ↓ decreased).

Sr. No.	Metabolite	p.value	FDR	Tukey's HSD	CovP/ Control	CAP/ Control
1	Aspartic acid	2.65E-04	7.65E-04	CovP-Control	↑	
2	RI1637.76	5.01E-06	2.89E-05	CovP-Control	↑	
3	RI3819.77	5.66E-06	3.14E-05	CovP-Control	↑	
4	RI2591.08	2.15E-05	1.01E-04	CovP-Control	↓	
5	RI3790.91	2.83E-05	1.29E-04	CovP-Control	↑	
6	Glycine	7.04E-05	2.64E-04	CovP-Control	↑	
7	Serine	2.25E-04	6.89E-04	CovP-Control	↑	
8	RI1039.49	2.54E-04	7.47E-04	CovP-Control	↓	
9	RI3110.71	2.14E-03	5.10E-03	CovP-Control	↓	
10	RI1992.66	1.54E-10	4.38E-09	CovP-Control; CovP-CAP	↑	
11	Lactic acid	5.44E-08	5.10E-07	CovP-Control; CovP-CAP	↑	
12	Taurine	1.72E-07	1.29E-06	CovP-Control; CovP-CAP	↑	
13	Pyroglutamic acid	3.83E-07	2.61E-06	CovP-Control; CovP-CAP	↑	
14	RI2719.45	4.01E-06	2.49E-05	CovP-Control; CovP-CAP	↓	
15	RI1503.85	2.16E-05	1.01E-04	CovP-Control; CovP-CAP	↓	

16	RI1317.28	1.11E-04	3.71E-04	CovP-Control; CovP-CAP	↓	
17	RI1854.02	3.28E-04	8.95E-04	CovP-Control; CovP-CAP	↓	
18	RI1125.72	2.40E-04	7.21E-04	CovP-CAP		
19	Fumaric acid	2.36E-10	4.40E-09	CovP-Control; CAP-Control	↓	↓
20	Phenylalanine	3.68E-10	5.52E-09	CovP-Control; CAP-Control	↑	↑
21	N-Carboxyglycine	5.41E-10	7.38E-09	CovP-Control; CAP-Control	↓	↓
22	RI2109.26	3.14E-05	1.39E-04	CovP-Control; CAP-Control	↑	↑
23	RI1619.34	3.31E-05	1.42E-04	CovP-Control; CAP-Control	↑	↑
24	RI1532.53	8.60E-05	3.07E-04	CovP-Control; CAP-Control	↑	↑
25	Ornithine	2.09E-04	6.60E-04	CovP-Control; CAP-Control	↑	↑
26	RI1759.09	2.73E-04	7.72E-04	CovP-Control; CAP-Control	↑	↑
27	Galactose	6.01E-04	1.56E-03	CovP-Control; CAP-Control	↑	↑
28	Lyxose	1.02E-03	2.56E-03	CovP-Control; CAP-Control	↑	↑
29	RI1201.11	2.30E-03	5.30E-03	CovP-Control; CAP-Control	↑	
30	RI1451.15	7.76E-15	1.16E-12	CAP-Control; CovP-CAP; CovP-Control	↑	↑
31	RI1467.82	2.57E-11	1.93E-09	CAP-Control; CovP-CAP; CovP-Control	↑	↑
32	Maltose	1.75E-10	4.38E-09	CAP-Control; CovP-CAP; CovP-Control	↑	↑
33	RI2647.41	2.64E-10	4.40E-09	CAP-Control; CovP-CAP; CovP-Control	↑	↑
34	Ribose	1.44E-08	1.54E-07	CAP-Control; CovP-CAP; CovP-Control	↑	↑
35	Threonic acid	4.65E-11	2.33E-09	CAP-Control; CovP-CAP		↑
36	Xylitol	1.33E-10	4.38E-09	CAP-Control; CovP-CAP		↑
37	RI1857.13	2.27E-10	4.40E-09	CAP-Control; CovP-CAP		↑
38	meso-Erythritol	3.52E-09	4.41E-08	CAP-Control; CovP-CAP		↑
39	RI1680.27	1.20E-08	1.39E-07	CAP-Control; CovP-CAP		↑
40	RI1633.64	2.60E-08	2.60E-07	CAP-Control; CovP-CAP		↑
41	RI2340.12	6.12E-08	5.40E-07	CAP-Control; CovP-CAP		↑
42	RI1360.57	1.08E-07	9.02E-07	CAP-Control; CovP-CAP		↑
43	Urea	1.17E-07	9.20E-07	CAP-Control; CovP-CAP		↑
44	RI2960.53	3.38E-07	2.41E-06	CAP-Control; CovP-CAP		↑
45	RI2855.95	1.64E-06	1.07E-05	CAP-Control; CovP-CAP		↑
46	RI1361.34	4.15E-06	2.49E-05	CAP-Control; CovP-CAP		↓
47	RI1612.75	9.45E-06	4.94E-05	CAP-Control; CovP-CAP		↑
48	Fructose	9.56E-06	4.94E-05	CAP-Control; CovP-CAP		↑
49	RI2509.680	1.47E-05	7.33E-05	CAP-Control; CovP-CAP		↓
50	RI1373	3.60E-05	1.50E-04	CAP-Control; CovP-CAP		↑
51	Glucuronic acid	4.11E-05	1.67E-04	CAP-Control; CovP-CAP		↑
52	RI1150.81	4.57E-05	1.80E-04	CAP-Control; CovP-CAP		↑
53	RI1390.85	9.36E-05	3.26E-04	CAP-Control; CovP-CAP		↑
54	Gluconic acid	1.10E-04	3.71E-04	CAP-Control; CovP-CAP		↑
55	RI1083.74	1.55E-04	5.04E-04	CAP-Control; CovP-CAP		↑
56	Gluconic acid 1,5-lactone	2.11E-04	6.60E-04	CAP-Control; CovP-CAP		↑
57	Erythrose-4-phosphate	6.26E-04	1.59E-03	CAP-Control; CovP-CAP		↑
58	RI1383.17	1.87E-03	4.51E-03	CAP-Control; CovP-CAP		↑
59	RI1261.62	2.18E-03	5.12E-03	CAP-Control; CovP-CAP		↑
60	RI2812.75	3.26E-04	8.95E-04	CAP-Control		↑
61	RI2603.98	3.75E-04	1.00E-03	CAP-Control		↑
62	Methionine	5.16E-04	1.36E-03	CAP-Control		↑
63	Tyrosine	1.19E-03	2.93E-03	CAP-Control		↑
64	RI1782.79	2.81E-03	6.38E-03	CAP-Control		↑
65	RI3283.77	3.53E-03	7.91E-03	CAP-Control		↑
66	Isoleucine	4.13E-03	8.98E-03	CAP-Control		↑

Table S4. List of all cytokines with their means of delta values, standard deviation and median.

Cytokines	Groups	Mean	Standard Deviation	Median	Range
Alb_delta	Control	-0.0909	2.0681	-1.0000	8.0000
	Pneumonia	2.4118	8.1705	1.0000	35.0000
	COVID-19	2.1111	7.7208	2.0000	22.0000
GEW_delta	Control	0.1905	3.6826	1.0000	13.0000
	Pneumonia	3.0500	14.4858	-1.0000	66.0000
	COVID-19	3.3333	15.2807	-1.0000	44.0000
Adiponectin_delta	Control	-0.0840	0.8214	0.1000	3.2000
	Pneumonia	0.0957	1.2608	-0.1000	5.0000

	COVID-19	-0.1444	0.4362	-0.1000	1.5000
AAT_delta	Control	-0.0257	0.4075	-0.1000	1.6000
	Pneumonia	0.0050	0.8763	-0.1500	3.6000
	COVID-19	-0.4222	0.7710	-0.5000	2.3000
A2Macro_delta	Control	0.0455	0.6139	0.0500	2.4000
	Pneumonia	-0.2227	0.7597	-0.1500	3.1000
	COVID-19	0.0789	0.2983	0.2000	1.0000
Lp(a)_delta	Control	-6.0591	32.4131	0.8500	146.0000
	Pneumonia	-29.8182	110.0972	-2.5000	582.0000
	COVID-19	4.0000	23.9344	-4.0000	74.0000
B2M_delta	Control	-0.0450	0.4582	-0.1000	2.0000
	Pneumonia	-0.3100	2.1094	-0.2000	10.0000
	COVID-19	-0.3000	0.4989	-0.2500	1.8000
BDNF_delta	Control	-0.2684	2.2774	-0.5000	10.5000
	Pneumonia	0.2589	1.5914	0.2000	6.8000
	COVID-19	0.3650	0.9493	0.4000	2.7000
CRP_delta	Control	-2.0108	9.4620	-0.1350	46.0000
	Pneumonia	-7.6957	34.0634	-4.0000	165.0000
	COVID-19	-24.6000	70.9322	-22.5000	267.0000
C3_delta	Control	-0.0319	0.2556	-0.0200	1.1300
	Pneumonia	-0.0668	0.2218	-0.1000	0.8000
	COVID-19	-0.0378	0.1861	-0.1000	0.4500
EN-RAGE_delta	Control	-4.7692	125.2234	-5.5000	620.0000
	Pneumonia	-123.2609	238.6603	-85.0000	968.0000
	COVID-19	-469.2000	496.6344	-307.0000	1445.0000
Eotaxin-1_delta	Control	-15.4211	58.2297	-13.0000	217.0000
	Pneumonia	4.6667	71.7910	-14.0000	258.0000
	COVID-19	-15.8571	79.2095	-21.0000	204.0000
Factor VII_delta	Control	0.1200	25.7881	1.0000	92.0000
	Pneumonia	-4.1739	37.9792	-2.0000	176.0000
	COVID-19	-16.3000	21.4012	-15.5000	66.0000
FRTN_delta	Control	24.8400	169.2229	21.0000	930.0000
	Pneumonia	81.1739	323.1415	48.0000	1660.0000
	COVID-19	-246.5556	631.3195	-220.0000	2040.0000
Fibrinogen_delta	Control	-0.0032	0.0070	-0.0035	0.0240
	Pneumonia	-0.0091	0.0421	0.0010	0.1860
	COVID-19	-0.0046	0.0098	-0.0040	0.0270
Haptoglobin_delta	Control	0.0598	0.5508	0.0600	2.2000
	Pneumonia	-0.2229	0.7943	0.1000	3.0000
	COVID-19	-0.2875	2.1424	-0.2000	7.0000
IgA_delta	Control	-0.0542	0.5388	-0.0500	2.6000
	Pneumonia	-0.2025	0.4797	-0.2000	1.6000
	COVID-19	-0.5444	0.6064	-0.3000	1.7000
IgM_delta	Control	0.0295	0.3385	0.1000	1.2000
	Pneumonia	-0.0273	0.4278	-0.1000	2.0000
	COVID-19	-0.3889	0.4400	-0.3000	1.6000
ICAM-1_delta	Control	-0.3600	33.1145	4.0000	159.0000
	Pneumonia	-12.1739	75.7692	-4.0000	375.0000
	COVID-19	-105.7778	167.2586	-17.0000	449.0000
IL-1 beta_delta	Control	0.2056	3.5541	0.8000	11.7000
	Pneumonia	0.2167	3.0438	0000	10.0000
	COVID-19	-1.7222	2.1592	-1.6000	6.1000
IL-1RA_delta	Control	21.2400	126.3973	4.0000	682.0000
	Pneumonia	-39.9524	81.8227	-25.0000	402.0000
	COVID-19	-106.3000	122.5698	-87.5000	370.0000
IL-5_delta	Control	-8.5000	.	-8.5000	0000
	Pneumonia	-3.0000	5.6569	-3.0000	8.0000
	COVID-19	.	.	.	.
IL-6_delta	Control	-3.1545	5.4257	-5.0000	15.4500
	Pneumonia	-2.0263	7.8304	-1.0000	39.0000
	COVID-19	-20.2000	38.8009	-3.0000	127.0000
IL-8_delta	Control	1.3792	13.9171	-2.0000	74.0000
	Pneumonia	-0.6818	14.2641	1.0000	62.0000

	COVID-19	-7.2222	23.7370	4.0000	73.0000
IL-10_delta	Control	-2.7000	4.4981	-2.5000	12.0000
	Pneumonia	1.1667	5.2496	2.0000	18.0000
	COVID-19	-4.6000	7.2449	-3.5000	26.0000
IL-12p40_delta	Control	-0.0415	0.1762	-0.0900	0.6900
	Pneumonia	0.0323	0.1389	0.0500	0.5200
	COVID-19	0.0075	0.2193	-0.0050	0.6700
IL-17_delta	Control	0.1750	1.9276	0.3500	4.2000
	Pneumonia	0.0714	1.6469	0.5000	4.3000
	COVID-19	-0.0333	2.8290	1.6000	4.9000
IL-18_delta	Control	-5.5000	91.2159	-9.5000	498.0000
	Pneumonia	-4.8333	100.3418	-6.0000	410.0000
	COVID-19	-95.0000	203.4939	-51.0000	670.0000
MIP-1 alpha_delta	Control	12.4167	24.9780	16.0000	75.0000
	Pneumonia	-13.1250	30.3268	-14.5000	118.0000
	COVID-19	4.0000	23.8956	9.0000	47.0000
MIP-1 beta_delta	Control	-15.7500	69.4258	-28.5000	239.0000
	Pneumonia	-11.0000	84.7899	-9.0000	340.0000
	COVID-19	-53.8000	97.0358	-58.0000	285.0000
MMP-3_delta	Control	-0.1044	0.9194	-0.1000	5.0000
	Pneumonia	-0.3162	1.7095	-0.5000	7.0000
	COVID-19	-0.3875	0.4883	-0.3000	1.4000
MMP-9_delta	Control	-5.3684	19.8248	-5.0000	65.0000
	Pneumonia	3.2500	21.0285	9.0000	69.0000
	COVID-19	5.8333	18.8087	-1.5000	44.0000
MCP-1_delta	Control	-22.1000	81.6369	-6.0000	292.0000
	Pneumonia	-6.6818	163.8697	45.5000	540.0000
	COVID-19	-134.2000	561.4406	-175.0000	2120.0000
Myoglobin_delta	Control	1.8619	13.3963	-1.0000	59.0000
	Pneumonia	0.3571	10.9376	1.0000	42.0000
	COVID-19	0.2500	3.2016	-1.0000	7.0000
PAI-1_delta	Control	-8.5652	39.7479	1.0000	150.0000
	Pneumonia	32.7391	63.1724	24.0000	267.0000
	COVID-19	-19.1000	49.7381	-27.5000	166.0000
PARC_delta	Control	-9.2400	62.2356	4.0000	326.0000
	Pneumonia	30.5652	119.0633	6.0000	665.0000
	COVID-19	-1.3000	22.4155	-11.0000	67.0000
SAP_delta	Control	-0.4522	2.9285	-0.7000	14.0000
	Pneumonia	-0.6048	3.2358	0.3000	14.0000
	COVID-19	-1.4444	2.6977	-2.0000	7.0000
SCF_delta	Control	-6.1522	84.7455	26.0000	328.0000
	Pneumonia	-25.6053	129.7365	36.0000	478.0000
	COVID-19	-68.5000	145.2810	-26.0000	359.0000
RANTES_delta	Control	-2.6043	9.5327	-2.0000	41.0000
	Pneumonia	-1.6186	6.5778	-1.0000	30.8000
	COVID-19	0.2000	3.8178	-1.0000	10.3000
TBG_delta	Control	0.0455	6.9862	2.0000	32.0000
	Pneumonia	-1.0909	8.0054	0000	30.0000
	COVID-19	-5.7778	5.9954	-5.0000	20.0000
TIMP-1_delta	Control	-6.2308	47.2130	3.0000	235.0000
	Pneumonia	18.5217	81.1440	14.0000	425.0000
	COVID-19	18.7000	153.8318	-23.5000	594.0000
TNF-alpha_delta	Control	1.0000	.	1.0000	0000
	Pneumonia	33.5000	0000	33.5000	0000
	COVID-19	-30.5000	.	-30.5000	0000
TNFR2_delta	Control	-4.6625	25.1806	-0.4000	136.0000
	Pneumonia	1.5652	9.2826	3.0000	38.0000
	COVID-19	3.7800	24.3492	-2.6000	89.0000
VCAM-1_delta	Control	-1.3846	168.9885	2.0000	791.0000
	Pneumonia	75.4762	233.0278	13.0000	1080.0000
	COVID-19	-5.0000	138.3962	-20.0000	468.0000
VEGF_delta	Control	-1.0435	59.4623	6.0000	259.0000
	Pneumonia	29.0476	79.9015	8.0000	349.0000

	COVID-19	-24.0000	92.2870	-45.0000	351.0000
VDBP_delta	Control	-4.2000	38.7933	-8.0000	178.0000
	Pneumonia	2.2609	37.5018	3.0000	159.0000
	COVID-19	-8.6000	37.5949	-3.5000	127.0000
vWF_delta	Control	-13.1200	65.5968	-8.0000	272.0000
	Pneumonia	-21.2727	176.8785	8.5000	1021.0000
	COVID-19	-64.8000	130.1109	-50.0000	479.0000
AFP_delta	Control	-0.0820	0.7295	-0.4000	1.4200
	Pneumonia	0.1318	0.8746	0.7100	2.5200
	COVID-19	0.7100	.	0.7100	0000
ANG-1_delta	Control	0.3846	10.1354	0000	47.0000
	Pneumonia	1.7762	11.6924	1.0000	56.0000
	COVID-19	-3.3889	4.7551	-2.0000	16.0000
AXL_delta	Control	0.4114	1.0057	0.2500	3.5000
	Pneumonia	-0.1417	1.0647	-0.2500	4.0000
	COVID-19	0.0875	1.2100	0.1000	4.2000
CA-125_delta	Control	-2.7500	7.0373	-2.0000	22.1500
	Pneumonia	-2.0275	12.0045	2.1000	47.0000
	COVID-19	1.8000	6.4285	2.4500	15.6000
CA-19-9_delta	Control	1.3955	6.9721	0000	27.0000
	Pneumonia	-1.0912	8.1036	-1.0000	36.0000
	COVID-19	-1.6188	6.1709	-2.4250	21.0000
CA-9_delta	Control	0.0151	0.1026	-0.0001	0.5300
	Pneumonia	-0.0053	0.0356	0.0100	0.1200
	COVID-19	0.0004	0.0567	-0.0235	0.1600
CEA_delta	Control	-0.2347	1.0656	-0.1000	4.5100
	Pneumonia	-0.1232	0.5443	-0.1000	2.5000
	COVID-19	0.0350	0.6395	-0.1000	2.1000
HCC-4_delta	Control	0.0318	0.3969	0.1000	1.6000
	Pneumonia	0.0700	0.6219	-0.1000	2.0000
	COVID-19	0.1171	0.1472	0.1000	0.4400
Decorin_delta	Control	0.0517	0.2130	0.1000	0.9000
	Pneumonia	-0.0353	0.3278	-0.1000	1.4000
	COVID-19	0.0143	0.3237	-0.2000	0.8000
FAS_delta	Control	-1.2667	4.5906	-2.000	20.6000
	Pneumonia	0.1000	7.5247	-0.5000	23.0000
	COVID-19	4.2700	16.2882	1.5000	63.0000
HGF_delta	Control	-0.5304	1.8497	-0.8000	8.0000
	Pneumonia	-2.8500	7.4527	-2.5000	36.0000
	COVID-19	-9.2556	29.6102	-3.0000	101.0000
hCG_delta	Control	-0.3000	.	-0.3000	0000
	Pneumonia	-0.0875	1.6555	0.3500	3.8500
	COVID-19	0.6667	2.0306	-0.3000	3.7000
IL-18bp_delta	Control	0000	1.1662	-0.1000	4.0000
	Pneumonia	-0.6923	3.9872	-1.0000	13.0000
	COVID-19	-0.1900	2.6510	-1.0000	8.0000
MMP-1_delta	Control	-0.6042	2.8472	0.0500	14.0000
	Pneumonia	-0.6000	10.1490	1.0000	52.0000
	COVID-19	-0.4100	5.0479	-1.5000	18.0000
MMP-7_delta	Control	0.0840	1.2912	0.2000	7.0000
	Pneumonia	-0.0833	1.1346	-0.1000	4.0000
	COVID-19	-0.4286	3.2201	-0.2000	10.4000
MMP-9, total_delta	Control	-69.7692	335.2541	-21.0000	1726.0000
	Pneumonia	-57.6957	204.1705	-56.0000	987.0000
	COVID-19	-269.2000	411.2982	-165.5000	1380.0000
NSE_delta	Control	0.4152	1.3924	0.0200	7.3100
	Pneumonia	-0.0690	0.3967	-0.1000	1.5900
	COVID-19	-0.1522	2.0987	-0.3000	7.6100
PECAM-1_delta	Control	-0.2917	7.7487	2.0000	35.0000
	Pneumonia	-2.3333	14.1751	2.0000	65.0000
	COVID-19	-1.8889	10.5883	-5.0000	32.0000
SP-D_delta	Control	-0.2125	1.8319	-0.1000	9.0000
	Pneumonia	-2.9476	7.3474	-1.0000	35.0000

	COVID-19	0.5556	9.1667	-2.0000	30.0000
TRAIL-R3_delta	Control	0.1261	0.9117	0.1000	3.7000
	Pneumonia	-0.7667	2.6337	-1.0000	12.0000
	COVID-19	-0.4222	0.9378	-0.6000	3.1000
